# Survival-Critical Genes Associated with Copy Number Alterations in Lung Adenocarcinoma

**DOI:** 10.3390/cancers13112586

**Published:** 2021-05-25

**Authors:** Chinthalapally V. Rao, Chao Xu, Mudassir Farooqui, Yuting Zhang, Adam S. Asch, Hiroshi Y. Yamada

**Affiliations:** 1 Center for Cancer Prevention and Drug Development, Department of Medicine, Hematology/Oncology Section, University of Oklahoma Health Sciences Center (OUHSC), Oklahoma City, OK 73104, USA; yuting-zhang@ouhsc.edu; 2 Stephenson Cancer Center, University of Oklahoma Health Sciences Center (OUHSC), Oklahoma City, OK 73104, USA; adam-asch@ouhsc.edu; 3 VA Medical Center, Oklahoma City, OK 73104, USA; 4 Hudson College of Public Health, University of Oklahoma Health Sciences Center (OUHSC), Oklahoma City, OK 73104, USA; chao-xu@ouhsc.edu; 5 Department of Neurology, University of Iowa Hospitals and Clinics, Iowa City, IA 52242, USA; mudassir-farooqui@uiowa.edu

**Keywords:** genomic instability, lung tumor, tumor heterogeneity, tumor genomics

## Abstract

**Simple Summary:**

Genomic instability affects cancer evolution and impacts carcinogenesis, therapy response, recurrence/prognosis, thus overall clinical outcomes. To comprehensively identify genes and pathways affecting genomic instability, we employed a novel data-mining strategy (Gene Expression to Copy Number Alterations; “GE-CNA” approach) and identified 1578 genes whose expression associates with Copy Number Alterations in human lung adenocarcinoma. Among the 1578 genes, we identified 39 as survival-critical. They represent potential targets for therapy development.

**Abstract:**

Chromosome Instability (CIN) in tumors affects carcinogenesis, drug resistance, and recurrence/prognosis. Thus, it has a high impact on outcomes in clinic. However, how CIN occurs in human tumors remains elusive. Although cells with CIN (i.e., pre/early cancer cells) are proposed to be removed by apoptosis and/or a surveillance mechanism, this surveillance mechanism is poorly understood. Here we employed a novel data-mining strategy (Gene Expression to Copy Number Alterations [CNA]; “GE-CNA”) to comprehensively identify 1578 genes that associate with CIN, indicated by genomic CNA as its surrogate marker, in human lung adenocarcinoma. We found that (a) amplification/insertion CNA is facilitated by over-expressions of DNA replication stressor and suppressed by a broad range of immune cells (T-, B-, NK-cells, leukocytes), and (b) deletion CNA is facilitated by over-expressions of mitotic regulator genes and suppressed predominantly by leukocytes guided by leukocyte extravasation signaling. Among the 39 CNA- and survival-associated genes, the purine metabolism (PPAT, PAICS), immune-regulating CD4-LCK-MEC2C and CCL14-CCR1 axes, and ALOX5 emerged as survival-critical pathways. These findings revealed a broad role of the immune system in suppressing CIN/CNA and cancer development in lung, and identified components representing potential targets for future chemotherapy, chemoprevention, and immunomodulation approaches for lung adenocarcinoma.

## 1. Introduction

Tumor genomics data have revealed a wide variety of tumor heterogeneity, the underlying driver of which is genomic instability. There are two major modes of genomic instability: Microsatellite Instability (MIN) and Chromosome Instability (CIN). Although not mutually exclusive, MIN is generally associated with functional alteration in proteins involved in DNA replication and repair, while CIN is mainly associated with mitotic dysregulation. In a solid tumor, genomic instability, especially large-scale chromosomal alterations caused by CIN, can be indicated with Copy Number Alterations (CNA) as its surrogate indicator [1]. Meta analyses indicated that high CIN is a marker of poor prognosis in colorectal, breast and lung cancers [2,3], consistent with the notion that genomic instability aids tumor evolution and fitness for survival under the challenges of cancer therapies [4]. Transgenic mouse-based studies demonstrated that elevated CIN can cause various cancers, including sporadic tumors in lung and liver [5,6,7]. Induced high CIN in mice increases the risk of lung cancer recurrence [8]. Thus, CIN is involved in carcinogenesis, drug response, and cancer recurrence, influencing major aspects of cancer prevention and therapy in the clinic setting [9,10,11]. The notion to target CIN and/or aneuploidy has long been proposed.

However, genes that govern the degree of CIN and CNA in tumors have been difficult to identify in a comprehensive manner. As genes/pathways to “target” remain unclear, the “Targeting CIN” concept has not been efficiently translated in clinic. Some of the “frequently mutated genes” in a given cancer can also play a role in genomic instability, as observed in colon cancer [11]. Yet this is not always the case, or is hard to demonstrate, in tumors with few penetrating mutations. Since CIN-associated mitotic regulator genes, such as the spindle checkpoint components (e.g., Mad1, Mad2, BubR1), are not always heavily mutated in tumors with high CIN [12,13], the understanding of how tumor CIN occurs remains elusive. By comparing gene expression profiles of high CNA tumors and low CNA tumors (a CNA-GE approach), correlations between high mitotic regulator expression and low immune signature were demonstrated in 12 types of cancers [14]. However, this general correlation is yet to be translated to targeted therapies. As aneuploid effects are proposed to be highly organ-specific [15], detailed analysis of an organ of interest is needed to identify specific markers and pathways for therapeutic translation.

Based on the tumor development profile in CIN mouse models [5,6,7], we hypothesized that CIN most prominently affects tumor development in lung and liver.

Using a novel in-silico analysis covering all reported human genes, we set out to comprehensively identify human genes whose expression shows correlation with CNA in human lung adenocarcinoma (the gene expression to CNA; “GE-CNA” strategy), anticipating that this approach would directly illuminate the network of genes through which lung cancer develops high CIN, or the mechanism by which the body antagonizes lung cells with genomic instability.

## 2. Materials and Methods

We downloaded the Lung Adenocarcinoma (TCGA, PanCancer Atlas, 2018) datasets, including the gene expression profile (available number of samples *n* = 510), copy number alterations (*n* = 511), mutation from whole exome sequencing (*n* = 566), patients’ survival and clinical data (*n* = 566), from cBioportal (https://www.cbioportal.org/study/summary?id=luad_tcga_pan_can_atlas_2018 accessed 1 May 2020) [16,17]. The Z-scores of mRNA expression (batch normalized by RSEM [18] from Illumina HiSeq_RNASeqV2) were used. The putative copy-number alteration (CNA) was estimated from GISTIC 2.0 [19], with 0 indicating neutral/no change, positive value indicating gain/amplification, and negative value indicating deletion. We analyzed all CNAs, amplification CNAs, and deletion CNAs.

There were 20,531 genes and 510 subjects in the downloaded gene expression file. We excluded 340 genes that were completely missing in all subjects, while the included genes were complete in all subjects. For each gene, we sorted its expression in 510 subjects and picked the subjects with the top and bottom ten expression values to form a high expression group and a low expression group. We extracted the number of CNA of subjects in the high and low expression groups from the downloaded CNA file, from which missing values were excluded. We used Student’s *t*-test to examine the difference of CNA counts in two groups at significance level of 0.05. Multiple-testing was adjusted by *q*-value [20]. 

The significant genes were further divided into two groups: higher expression that resulted in more CNAs and higher expression that resulted in fewer CNAs. The gene set enrichment analyses were conducted by IPA (Ingenuity Pathway Analysis, QIAGEN Inc., https://www.qiagenbioinformatics.com/products/ingenuity-pathway-analysis) (Germantown, MD, USA) [21] at significance level of 0.05 after Benjamini–Hochberg correction [22]. The presented pathway graphs were generated by IPA as well.

The association between the gene alteration and the patients’ overall survival was examined by the Cox Proportional-Hazards Model, with adjustment of the patients’ age and tumor stage. The adjusted covariates were selected by their univariate Cox regression *p*-value < 0.05. All available variables were considered, such as age, race, and tumor stage. We combined race groups with small sample size and used race variable with two levels: White and Other. We combined sub-level of tumor stage under each stage of stage 1 to 4. The tumor stage with four levels was used in the analysis. Patients having missing value were excluded. The Hazard Ratio (HR) and *p*-value of the gene were reported. We followed the definition of “altered” subject in cBioportal: altered = CNA (type of high-level amplification or homozygous deletion) + mutation (from whole exome sequencing). If a subject has any CNA and mutation in the gene, the subject is defined as altered. Otherwise, the subject is defined as unaltered. The difference of gene expression level in the altered and unaltered groups was tested by the Wilcoxon rank sum test. The significance level was 0.05. Survival curves and boxplots by altered/unaltered group are presented accordingly. We implemented all statistical analyses using R (v4.0.3) and R packages.

## 3. Results

**Figure 1:**
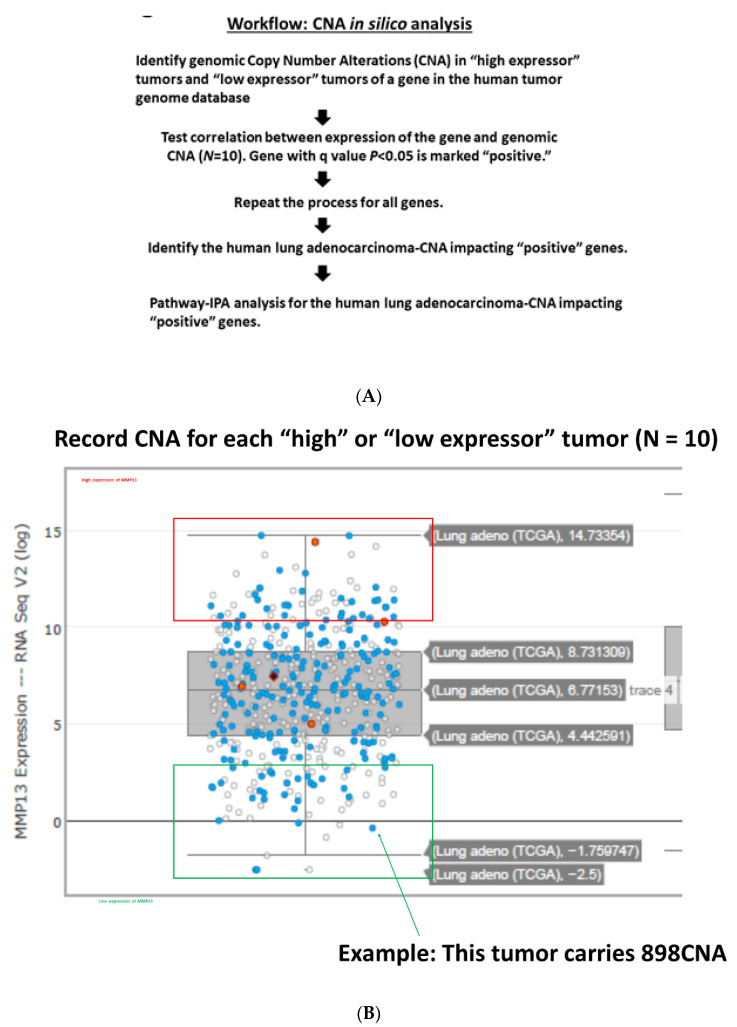
(**A**) For all genes, we recorded CNA for high expressor tumors (*N* = 10) and for low expressor tumors (*N* = 10). The CNA from the “high expressor” and “low expressor” groups were compared using unpaired *t*-test for each gene, testing the correlation between gene expression and numbers of CNA (*q*-value < 0.05). (**B**) An example of the process is shown with the MMP13 gene. We recorded CNA for each “high” or “low expressor” tumor (*N* = 10).

The analysis was performed as outlined in Figure 1A,B, aiming at identifying all genes whose expression levels show correlation to CNA with 95% confidence. This “GE-CNA” data-mining strategy initially indicated two groups of genes: (i) genes whose high expression correlates with a higher number of CNA (Figure S1A; Table S1), implicating the function as a facilitator of CIN and CNA, and (ii) genes whose high expression correlates with a lower number of CNA (Supplementary Figure S1B; Supplementary Table S2), implicating the function as a suppressor of CIN and CNA. Pathway-IPA analysis on the 492 group (i) CNA *facilitator* genes mapped their functions as mitotic regulator genes, including the Kinetochore Metaphase Signaling Pathway (Supplementary Figure S2A), Mitotic Roles of Polo-Like Kinase, Role of CHK Proteins in Cell Cycle Checkpoint Control, Cell Cycle: G2/M DNA Damage Checkpoint Regulation, Role of BRCA1 in DNA Damage Response, Hereditary Breast Cancer Signaling, Estrogen-mediated S-phase Entry, Protein Ubiquitination Pathway, ATM Signaling, Cell Cycle Control of Chromosomal Replication, and Mismatch Repair in Eukaryotes (Supplementary Table S1). In contrast, the functions of the 1086 group (ii) CNA *suppressor* genes were concentrated on immune functions, including the Th1 and Th2 Activation Pathway (Figure S2B), Complement System, Leukocyte Extravasation Signaling, Crosstalk between Dendritic Cells and Natural Killer Cells, Agranulocyte Adhesion and Diapedesis, Granulocyte Adhesion and Diapedesis, Th2 Pathway, Th1 Pathway, CTLA4 Signaling in Cytotoxic T Lymphocytes, Role of Pattern Recognition, Receptors in Recognition of Bacteria and Viruses, CD28 Signaling in T Helper Cells, and iCOS-iCOSL Signaling in T Helper Cells (Table S2). These results are in good agreement with Davoli et al. (2017), who employed the CNA-GE strategy (reverse of GE-CNA) and reported mitotic and immune signatures as traits of high or low CNA tumors.

For each gene, we tested the difference of clinical staging and gender distribution in included and non-included (control) group. Fisher’s exact test was used for categorical variable staging having 4 levels stage 1 to 4, while Chi-squared test was used for binary variable gender. After multiple testing correction by *q*-value, there are no significant differences in regards to clinical staging and gender (minimum adjusted *p*-value 0.095 and 0.452 respectively). In addition, we tested the difference of age using *t*-test. There are no significant differences in age.

**Figure 2:**
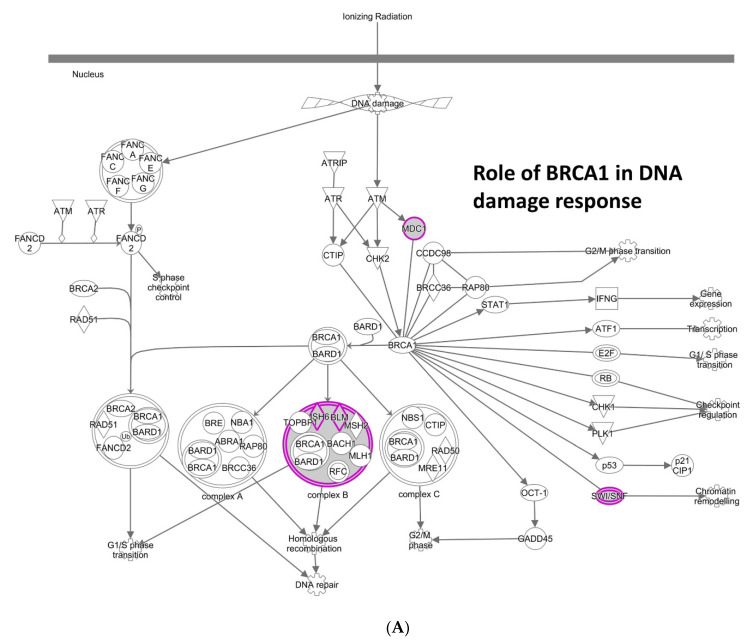

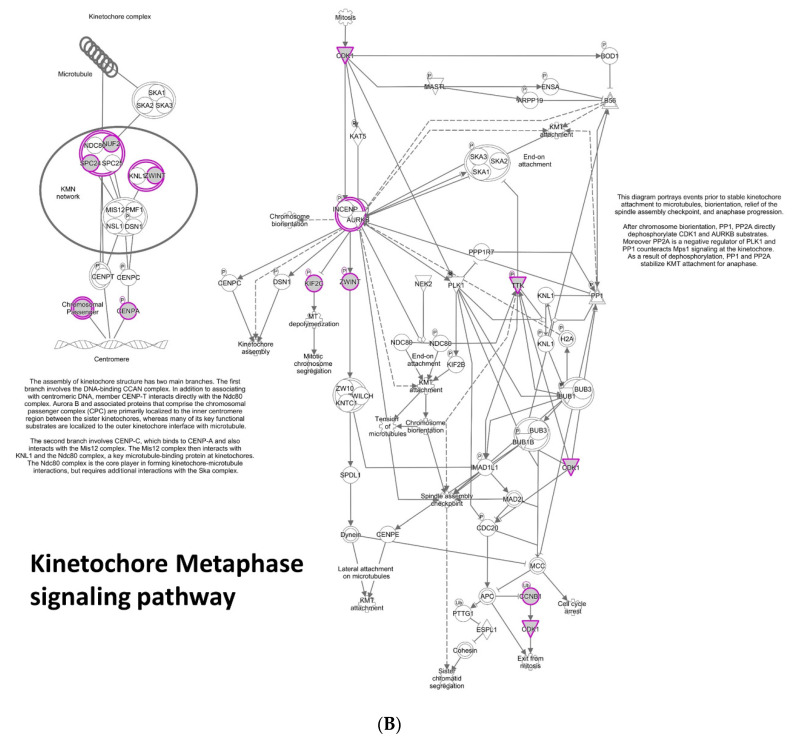
Different enrichments of CNA facilitator genes for amplification/insertion CNA and for deletion CNA (**A**) IPA for amplification/insertion CNA facilitator genes. Replication stress-inducing genes and pathways (e.g., Role of BRCA1 in DNA Damage Response) are enriched. (**B**) IPA for deletion CNA facilitator genes. Kinetochore Metaphase signaling pathway is shown. Purple highlighting indicates particular genes with significant GE-CNA correlations and/or a cluster of such genes in the IPA pathways.

The above initial analysis covered all CNA, both amplification/insertion and deletion. Next, we questioned whether amplification/insertion CNA and deletion CNA are differentially affected by different sets of genes. 161 genes in Table S3 [(iii) amplification/insertion CNA *facilitator* genes] were concentrated on the Role of BRCA1 in DNA Damage Response (Figure 2A), Hereditary Breast Cancer Signaling, and Cell Cycle Control of Chromosomal Replication, and included BLM, PHF10, CDC4, and CDK6. Their main functions are involved in the DNA replication and repair pathways; dysregulation causes MIN and/or DNA replication stress, which can lead to CIN [23]. Thus, amplification/insertion CNA is suggested to be predominantly driven by MIN or CIN caused by DNA replication stress. Individual genes known or unknown to affect genomic stability were also identified. For example, among the top eight high-significance genes (*q*-value < 0.005), dysregulation of KPNA2 (Karyopherin α2/Importin α) (*q* = 0.0009) has long been known to cause mitotic defects, and KPNA2 is reported as overexpressed in various cancers with poor prognosis [24]. A previously unidentified function of KLHL7 (Kelch Like Family Member 7) and KLHL11, components of the BCR (BTB-CUL3-RBX1) E3 ubiquitin ligase complex, in lung adenocarcinoma genomic instability was also suggested (*q* = 0.0003, 0.04, respectively). GINS1 (*q* = 0.0029) is a component of the GINS DNA replication initiation complex involved in reactivation from quiescence [25]. FAM126A (*q* = 0.0021) is downregulated by catenin, suggested to be a part of the beta-catenin/Lef signaling pathway. PRR19 was recently identified as a partner of cyclin-like CNTD1, and is required for timely DSB repair and the formation of crossover-specific recombination complexes [26].

187 genes in Supplementary Table S4 [(iv) deletion CNA *facilitator* genes] were enriched with known mitotic regulators Kinetochore Metaphase Signaling Pathway (Figure 2B), Protein Ubiquitination Pathway, Cyclins and Cell Cycle Regulation, and Cell Cycle: G2/M DNA Damage Checkpoint Regulation, indicating that overexpression of mitotic regulator genes leads to deletion CNA, which also confirms the role of mitotic mis-regulations in CNA. Notably, PSMD14 deubiquitinase (*q* = 3.63646 × 10^−8^), a subunit of 26S proteasome, was recently identified as upregulated in NSCLC [27]. Seven other 26S/20S proteasome subunits were isolated (*q* < 0.05; PSMD12, PSMB4, PSMA6, PSMB6, PSMC1, PSMD3, and PSMB3), illuminating the importance of the 26/20S proteasome integrity in lung adenocarcinoma genome maintenance. KIF18B (Kinesin Family Member 18B) (*q* = 0.0105) forms a complex with KIF2C (Kinesin Family Member 2C) (*q* = 0.0313) and constitutes the microtubule plus-end depolymerizing activity during mitosis [28]. ZWINT (ZW10 Interacting Kinetochore Protein) (*q* = 0.0126) is a kinetochore protein involved in mitotic checkpoint [29]. Other kinetochore proteins NUF2 (*q* = 0.0130) and SPC24 (*q* = 0.0244), as well as centromere proteins CENPI, CENPA, and CENPM, and mitotic regulatory kinases AURKA, TTK, and CDK1, are identified (*q* < 0.05). Also notable were transcription regulators (DPY30, MESP1, POU4F1, HOXC8, FOXK2, HOXC10, DLX5, E2F6, and SP8 [*q* < 0.05]). DPY30 (Dpy-30 Histone Methyltransferase Complex Regulatory Subunit) (*q* = 0.0109) is a core subunit of the SET1/MLL family of H3K4 methyltransferases that directly controls cell cycle regulators [30].

**Figure 3:**
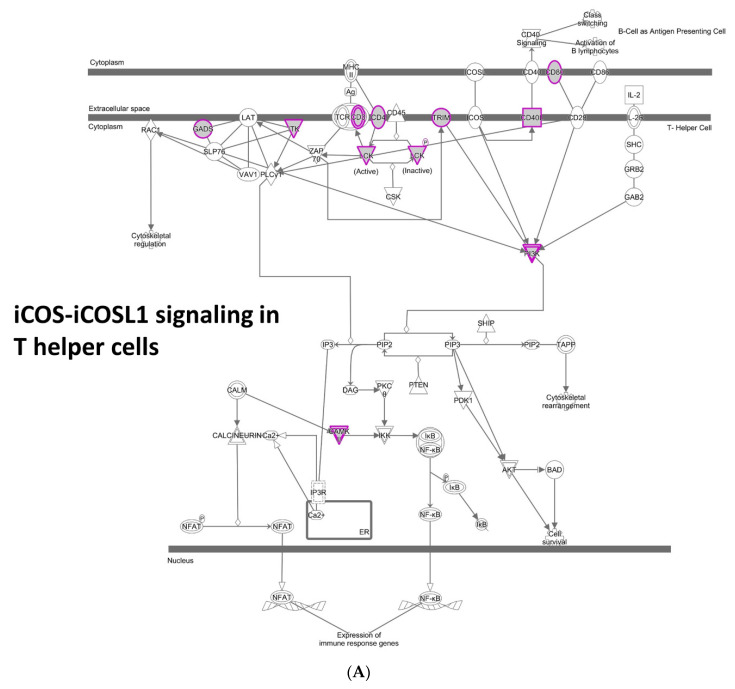

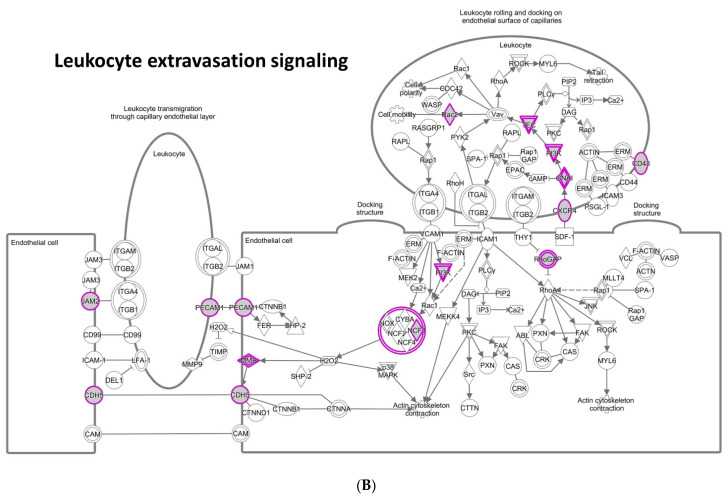
Different enrichments of CNA suppressor genes for amplification/insertion CNA and for deletion CNA (**A**) CNA suppressor genes for amplification/insertion CNA include a wide variety of immune pathways. e.g., iCOS-iCOSL signaling in T-helper cells. (**B**) CNA suppressor genes for deletion CNA showed specific enrichment (BH adjusted *p* = 0.0017) on Leukocyte Extravasation Signaling. The enrichment of Leukocyte Extravasation Signaling suggests that tumor cells with deletion CNA are recognized and removed by leukocytes guided by the signaling.

420 genes in Supplementary Table S5 [(v) amplification/insertion CNA *suppressor* genes] included a broad range of immune system genes. The IPA pathways involved include the iCOS-iCOSL Signaling in T Helper Cells (Figure 3A), Crosstalk between Dendritic Cells and Natural Killer Cells, Th1 and Th2 Activation Pathway, Systemic Lupus Erythematosus In B Cell Signaling Pathway, LPS/IL-1 Mediated Inhibition of RXR Function, Leukocyte Extravasation Signaling, Th2 Pathway, Th1 Pathway, Phenylethylamine Degradation I, and Xenobiotic Metabolism CAR Signaling Pathway. Among the top 36 high-significance genes (*q* < 0.005), GSTM5 (glutathione S transferase mu 5) (*q* = 0.0003) suggests the role of oxidative stress defense and detoxification in reducing tumor CNA. A decrease in GSTM5 was also observed in the sporadic lung cancer-prone genomic instability mouse model Sgo1^−/+^ [7]. Also notable are immunomodulatory surface receptors (CD37, CD22, LILRB3, TLR3) and other immune modulators (DPEP2, FGR, IL18) (*q* < 0.005).

In contrast, 407 genes in Supplementary Table S6 [(vi) deletion CNA *suppressor* genes] showed specific enrichment (BH adjusted *p*-value = 0.0017) on Leukocyte Extravasation Signaling (Figure 3B) including ARHGAP6, ARHGAP9, CDH5, CXCR4, GNAI2, ITGA1, ITK, JAM2, MMP19, NCF1, PECAM1, PIK3R5, RAC2, SPN, and TEC [31]. These genes control the movement of leukocytes from blood vessels towards the site of tissue damage or infection, suggesting that leukocytes guided by Leukocytes Extravasation Signaling may specifically target cells with deletion-CNA, which may be generated by overexpression of “deletion CNA *facilitator*” genes, or mitotic errors (Supplementary Table S4). In particular, the functions of the top 30 high-significance genes (*q* < 0.005) are concentrated on protection at the cell surface (MUC17, TSPAN3, TFF2, MUCL3) and membrane-based signal transduction (CHRM1, IL5RA, CDH5, TLR2, PTPRQ).

**Figure 4:**
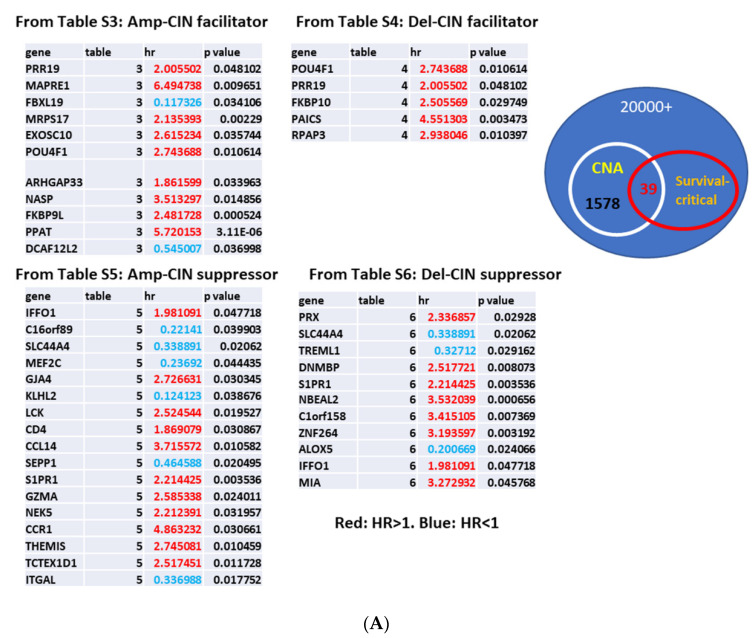

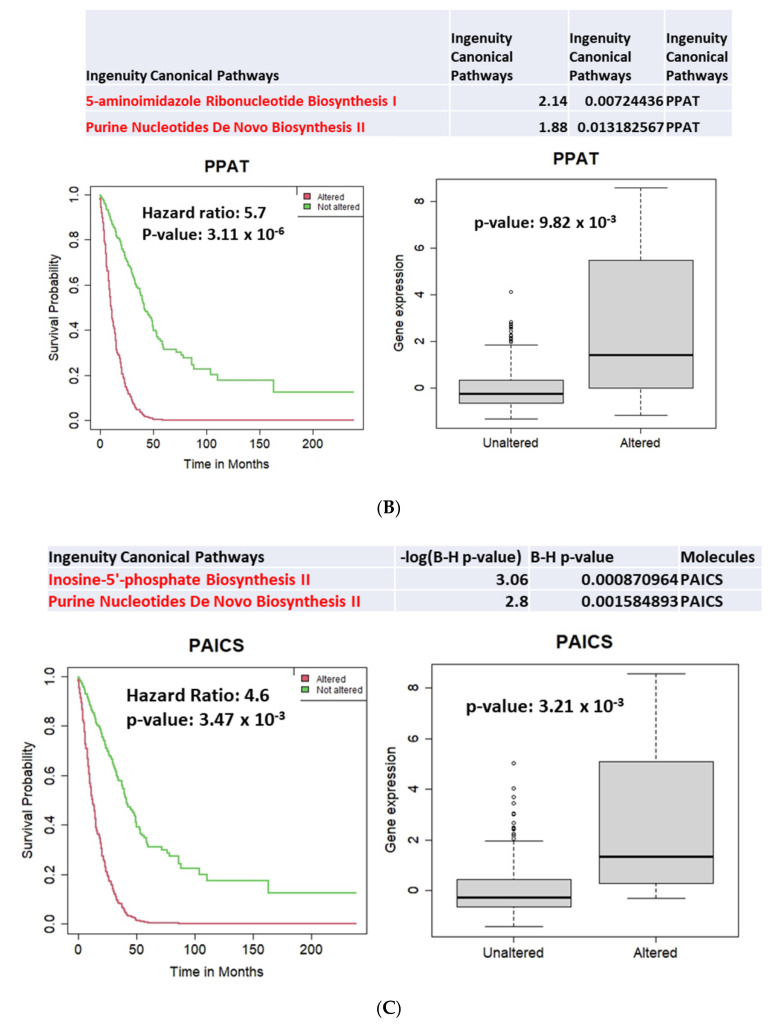

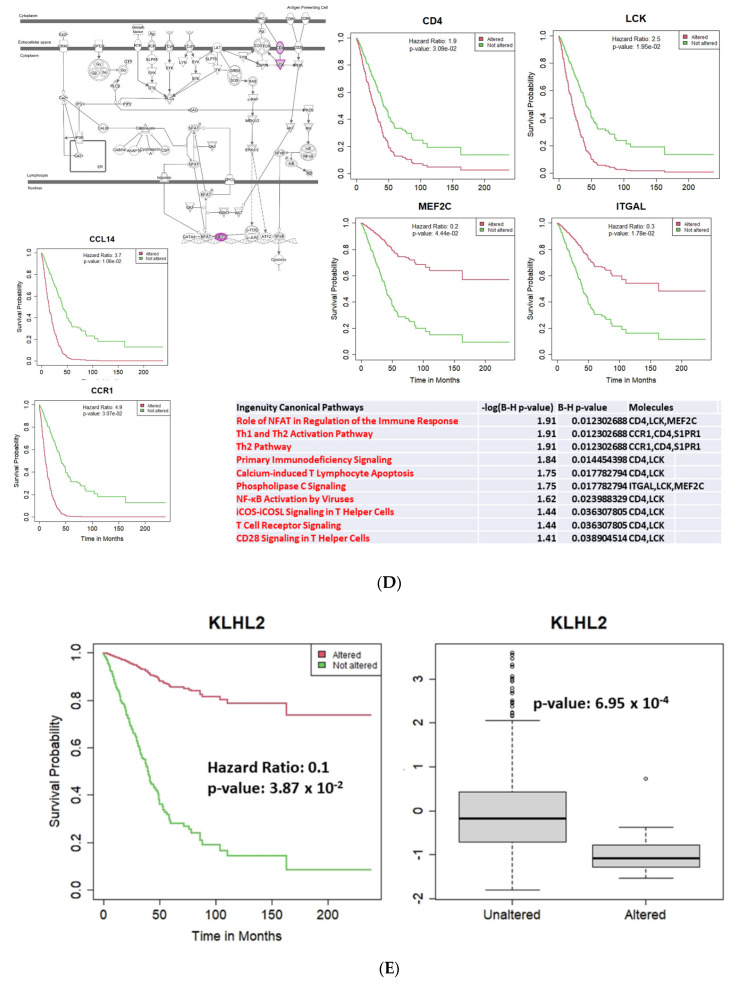

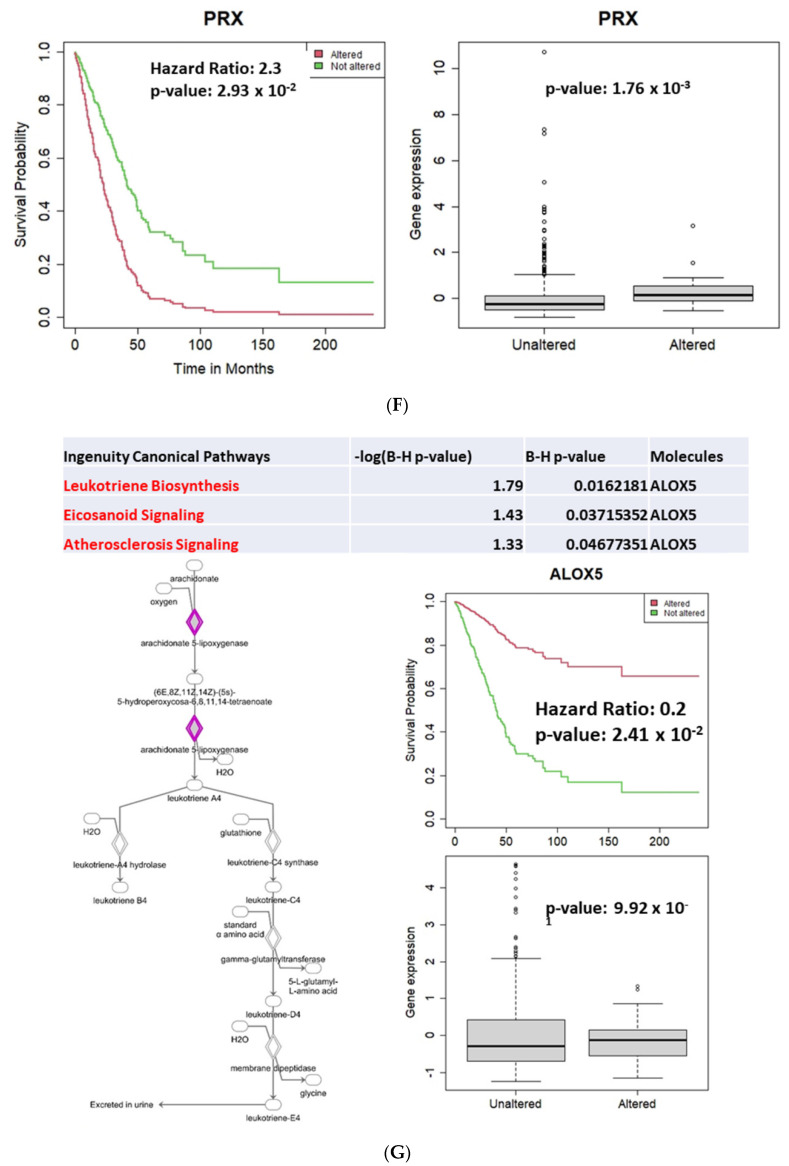
CNA facilitator/suppressor affecting patients’ survival (**A**) 39 genes for which expression levels correlate with both CNA and survival. Genes are shown indicating which category/Supplementary Table they are from. Highlighted in Red: HR > 1 (for which expression alterations increase risk). Blue: HR < 1. (**B**) PPAT overexpression (*p* = 0.0098) decreases survival (HR = 5.7, *p* = 3.11 × 10^−6^). PPAT (Phosphoribosyl Pyrophosphate Amidotransferase) catalyzes the first step of de novo purine nucleotide biosynthetic pathway. (**C**) PAICS overexpression (*p* = 0.0032) decreases survival (HR = 4.6, *p* = 0.0034). PAICS (Phosphoribosylaminoimidazole Carboxylase and Phosphoribosylaminoimidazolesuccinocarboxamide Synthase) belongs to the same purine biosynthetic pathway and catalyzing steps six and seven. (**D**) From many immune components, survival-critical 6 genes are enriched in CD4-LCK-MEF2C axis and CCL14-CCR1 axis, thus these two axes emerge as survival-critical pathways. Pathway analysis illuminates CD4-LCK-MEF2C axis and CCL14-CCR1 axis as critical pathways for patients’ survival. These two pathways may represent critical immunomodulation targets. (**E**) Decreased expression of KLHL2 (*p* = 6.95 × 10^−4^) is a marker for better prognosis (HR = 0.1, *p* = 0.0387). (**F**) Increased expression of PRX (Periaxin) (*p* = 0.0017) is a marker for poor prognosis (HR = 2.3, *p* = 0.0293). (**G**) ALOX5-mediated leukotriene biosynthesis plays a role in survival (HR = 0.2, *p* = 0.0241).

To identify potential targets for therapeutic drug or intervention, we surveyed the gene lists and tested whether the gene expression level affected patients’ overall survival, adjusted with covariates for lung adenocarcinoma. The second screening identified 39 genes for which expression alterations correlate with survival (Figure 4A; Supplementary Table S7. Among the 39 genes, 29 genes indicated a Hazard Ratio (HR) > 1, for which expression alterations increase risk, while 10 genes indicated HR < 1. Among CNA facilitators, PPAT and PAICS both indicated HR > 1 and their altered expressions were overexpression; both are involved in purine biosynthesis and metabolism (Figure 4B,C). Previous studies indicated that PPAT and PAICS were highly expressed in various cancers at advanced stages and involved in progression, which led to proposals for PPAT and PAICS as therapeutic inhibition targets [32]. Among CNA suppressors, the CD4-LCK-MEF2C axis and CCL14-CCR1 axis emerged as survival-critical pathways (Figure 4D). KLHL2 (Kelch Like Family Member 2; a ubiquitin ligase) indicated HR = 0.1, and decreased KLHL2 expression correlated with better prognosis (*p* = 0.0387) (Figure 4E). PRX (Periaxin, a key myelination protein) indicated HR = 2.3, and overexpression correlated with poor prognosis (*p* = 0.0293) (Figure 4F). These findings suggest that KLHL2 and/or PRX may be a novel inhibition target for therapeutic purposes. ALOX5/5-LOX (Arachidonate 5-lipoxygenase) emerged as del-CNA suppressor (HR = 0.2, *p* = 0.0241; Figure 4G).

## 4. Discussion

Previous works on cancer immunity or transcriptome had limited sample sizes and/or tested specific genes/pathways, e.g., [33,34]. The present study utilized 560 pooled non-overlapping lung adenocarcinoma data in the TCGA genome database and tested all 20,000+ human genes, and thus illustrates a GE landscape in an unbiased manner. This “gene expression to CNA (GE-CNA)” data-mining strategy has comprehensively uncovered 1578 genes associated with human lung adenocarcinoma CNA and genomic stability, and the pathways to which they belong. 492 CNA *facilitator* genes (Supplementary Table S1; Supplementary Figure S2A) and 1086 CNA *suppressor* genes (Supplementary Table S2; Supplementary Figure S2B) indicated a stark contrast in their functions, rather than simple loss- or gain-of-function. At the same time, the GE-CNA strategy results showed some similarities with the results of previous CNA-GE analysis [14].

To obtain additional mechanistic insights, we subcategorized the identified genes into four groups: 163 amplification/insertion CNA *facilitator* genes (Supplementary Table S3; Figure 2A); 187 deletion CNA *facilitator* genes (Supplementary Table S4; Figure 2B); 420 amplification/insertion CNA *suppressor* genes (Supplementary Table S5; Figure 3A); and 407 deletion CNA *suppressor* genes (Supplementary Table S6; Figure 3B). In theory, amplification/insertion CNA *facilitator* genes (Supplementary Table S3; Figure 2A) and amplification/insertion CNA *suppressor* genes (Supplementary Table S5; Figure 3A), and deletion CNA *facilitator* genes (Supplementary Table S4; Figure 2B) and deletion CNA *suppressor* genes (Supplementary Table S6; Figure 3B), are functionally antagonistic. The antagonistic relationship suggests that (a) amplification/insertion CNA associated with MIN or CIN with DNA replication stress can be suppressed by a broad range of immune cells (T, B, NK-cells, leukocytes), while (b) deletion CNA associated with mitotic regulator overexpression can be suppressed more specifically by leukocytes guided by leukocyte extravasation signaling. This prediction directs our attention to leukocytes in suppressing mitotic error-mediated “deletion CIN” tumor cells for designing novel modalities of immunotherapy in the future. CIN cancers respond to PD1/PD-L1 blockade immunotherapies poorly compared with MIN cancers [35], thus PD1/PD-L1 blockade therapies are approved for MIN cancers by the US FDA [36]. This phenomenon may be in part caused by the diversification of the immune pathways involved in targeting CIN and MIN.

Our discoveries are consistent with the notion that chromosome mis-segregation generates senescent cells with complex karyotypes/aneuploids and that they are eliminated by the immune system [15]. Yet, previous studies mainly reported innate characteristics of aneuploid cells that elicit immune activation, and only a limited number of immune cells or the trigger that responds to the activation signaling have been identified. For example, poly/hyperploid-type aneuploidy is sensed by immune cells with ER chaperone calreticulin, then eliminated by immunogenic death. Cells expressing NKG2D and DNAM1 ligands can be selectively targeted by NK cells [37,38]. This GE-CNA data-mining project successfully identified these pathways (Natural Killer Cell Signaling). Indeed, a majority of the CNA *suppressors* are immune signatures. The importance of immunomodulation in cancer therapy has been well established. Intervening PD1/PDL1 and CTLA4 pathways emerged as cancer immunotherapy modalities with efficacy and are being actively pursued [39]. Both pathways were identified (CTLA4 Signaling in Cytotoxic T Lymphocytes and PD-1, PD-L1 cancer immunotherapy pathway), as additional validation for this screening. Consistent with identification of the CD4-LCK-MEC2C axis, CD4 was identified as a key contributor to the PD1/PD-L1 immunotherapy efficacy, showing a critical role of CD4-mediated pathway in antagonizing cancer [40].

Newly illuminated with the present study and of particular clinical interest are survival-critical 39 genes and pathways of purine biosynthesis (PPAT, PAICS), immune-regulating CD4-LCK-MEC2C and CCL14-CCR1 axes, E3 ubiquitin ligase (KLHL2), PRX, and arachidonic acid metabolism ALOX5. In other organs (e.g., colon, pancreas), ALOX5 inhibition with COX1/2 inhibitors shows cancer chemoprevention effects [41,42,43]. In lung, ALOX5 may play a surveillance role for cells with CNA, as other researchers have suggested that ALOX5 inhibition can promote tumor development [44,45]. Overall, this study identified target genes and pathways for future cancer chemoprevention, therapy, and immunomodulatory approaches for improved patient survival.

This data-mining strategy may have missed some modalities of eliminating cells with genomic instability at an early phase, as the datasets used are for fully developed lung adenocarcinomas. For example, micronuclei-bearing mitotically-failed cells can activate the cGAS/STING pathway via cytosolic DNA [46,47,48], yet the cGAS/STING pathway did not appear. It can be presumed that the cGAS/STING pathway is involved in removal of very early-stage cancer or single micronuclei-bearing mitotically-failed cells, and the signaling may not be pronounced in lung adenocarcinoma in advanced stages. In the future, the use of datasets from early stage cancers or pre-cancerous lesions may provide information relevant to early cancer surveillance and prevention.

## 5. Conclusions

The GE-CNA strategy applied on human lung adenocarcinoma revealed 1578 genes whose expression level correlates with increased or decreased CNA. Pathway analysis on the genes suggests competitive and CNA type-specific relationships between CNA facilitator genes, mostly mitotic and cell cycle regulators, and suppressor genes, whose functions mainly involved in immune system thus likely consist of CNA immunosurveillance mechanisms. Secondary screening identified survival critical 39 genes among the 1578 genes. They represent potential targets for drug and/or immunomodulation approaches against lung adenocarcinoma.

## Data Availability

A public database (cbioportal.org) was utilized.

## Supplementary Materials

The following are available online at https://www.mdpi.com/article/10.3390/cancers13112586/s1, Figure S1: Results from a prototype/proof-of-principle study (AACR2019 Abstract #1199) Figure S2: CNA facilitators and CNA suppressors Table S1: CNA facilitator genes. Table S2: CNA suppressor genes. Table S3: Insertion/amplification CNA facilitator genes. Table S4: Deletion CNA facilitator genes. Table S5: Insertion/amplification CNA suppressor genes. Table S6: Deletion CNA suppressor genes. Table S7: Survival critical genes.
