# Supplementary material for: Survival-Critical Genes Associated with Copy Number Alterations in Lung Adenocarcinoma
Source: Cancers (Basel). 2021 May 25;13(11):2586. doi: 10.3390/cancers13112586 (PMC8197496; doi:10.3390/cancers13112586)
Supplement: Supplementary file 1 [file cancers-13-02586-s001.zip › cancers-1179863_supplementary.pdf]

cancers
MDPI

Article

Supplementary Material: Survival-Critical Genes Associated with Copy Number Alterations in Lung Adenocarcinoma

Chinthalapally V. Rao [1,2,3]*, Chao Xu [4], Mudassir Farooqui [5], Yuting Zhang [1], Adam S. Asch [2] and Hiroshi Y. Yamada [1,2]*

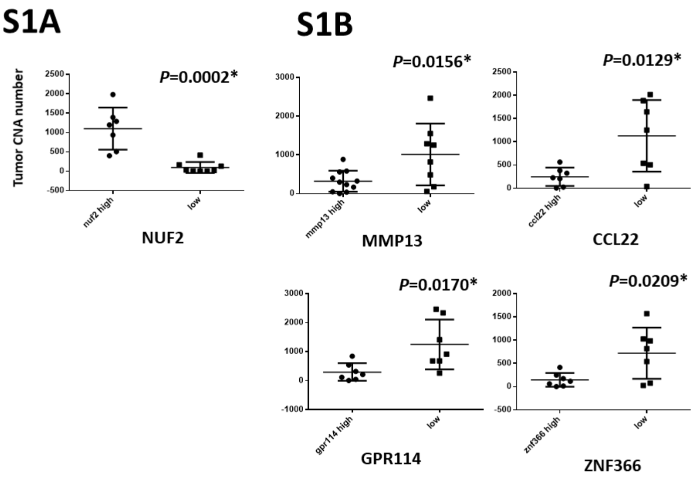

Figure S1. **(A)** An example of the group (i) *CNA facilitator* genes, Nuf2. High expression of NUF2 was correlated with high CNA. NUF2 is a kinetochore component, the effects of which on CIN were previously reported. [S1] **(B)** Examples of the group (ii) *CNA suppressor* genes from a prototype study (AACR2019 Abstract #1199).[S2] High expression of MMP13, CCL22, ZNF366, or GPR114 was correlated with low CNA.

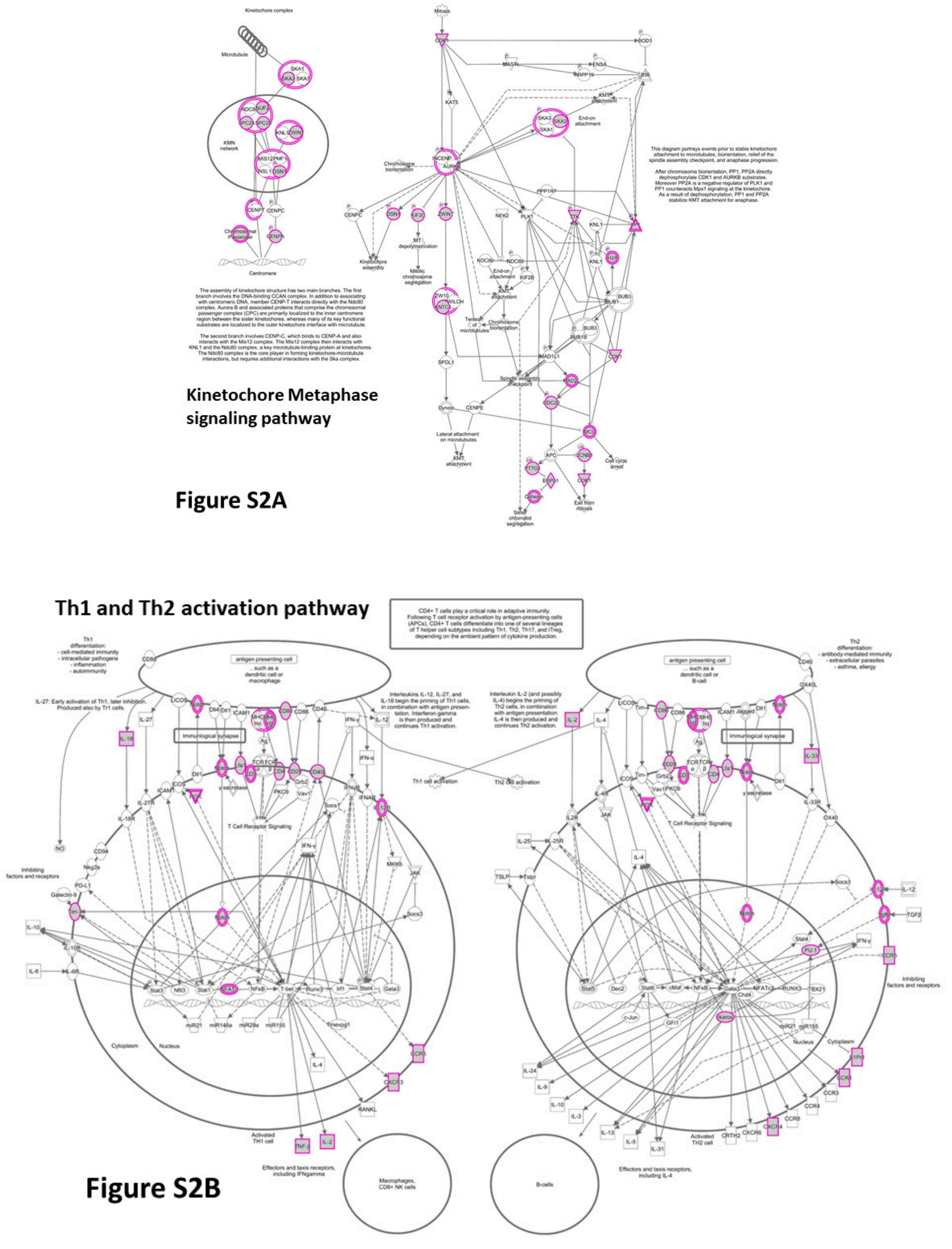

Figure S2. CNA facilitators and CNA suppressors (A) IPA for CNA facilitators; Kinetochore Metaphase Signaling Pathway. (B) IPA for CNA suppressors; Th1 and Th2 Activation Pathway. Genes identified in this screening are marked in purple.

References

S1

1. Liu et al. Integrated Genome-Wide Analysis of Gene Expression and DNA Copy Number Variations Highlights Stem Cell-Related Pathways in Small Cell Esophageal Carcinoma. *Stem Cells Int* 2018; 3481783. doi: 10.1155/2018/3481783

S2

2. Rao et al., Identification of candidate regulators of genomic instability in human lung adenocarcinoma through a new cross-species in silico analysis. *AACR2019* Abstract #1199. DOI: 10.1158/1538-7445.AM2019-1199

Table S7. CNA facilitator/suppressor affecting patients' survival 39 genes for which expression levels correlate with both CNA and survival. Genes are shown indicating which category/Supplementary Table they are from. Highlighted in Red: HR>1 (for which expression alterations increase risk). Blue: HR<1.

Figure S3. "Amp-CIN facilitator"

gene	table	hr	p value
PRR19	3	2.005502	0.048102
MAPRE1	3	6.494738	0.009651
FBXL19	3	0.117326	0.034106
MRPS17	3	2.135393	0.00229
EXOSC10	3	2.615234	0.035744
POU4F1	3	2.743688	0.010614
ARHGAP33	3	1.861599	0.033963
NASP	3	3.513297	0.014856
FKBP9L	3	2.481728	0.000524
PPAT	3	5.720153	3.11E-06
DCAF12L2	3	0.545007	0.036998

Figure S4. "Del-CIN facilitator".

gene	table	hr	p value
POU4F1	4	2.743688	0.010614
PRR19	4	2.005502	0.048102
FKBP10	4	2.505569	0.029749
PAICS	4	4.551303	0.003473
RPAP3	4	2.938046	0.010397

Figure S5. "Amp-CIN suppressor".

gene	table	hr	p value
IFFO1	5	1.981091	0.047718
C16orf89	5	0.22141	0.039903
SLC44A4	5	0.338891	0.02062
MEF2C	5	0.23692	0.044435
GJA4	5	2.726631	0.030345
KLHL2	5	0.124123	0.038676
LCK	5	2.524544	0.019527
CD4	5	1.869079	0.030867
CCL14	5	3.715572	0.010582
SEPP1	5	0.464588	0.020495
S1PR1	5	2.214425	0.003536
GZMA	5	2.585338	0.024011
NEK5	5	2.212391	0.031957
CCR1	5	4.863232	0.030661
THEMIS	5	2.745081	0.010459
TCTEX1D1	5	2.517451	0.011728
ITGAL	5	0.336988	0.017752

Figure S6. "Del-CIN suppressor".

gene	table	hr	p value
PRX	6	2.336857	0.02928
SLC44A4	6	0.338891	0.02062
TREML1	6	0.32712	0.029162
DNMBP	6	2.517721	0.008073
S1PR1	6	2.214425	0.003536
NBEAL2	6	3.532039	0.000656
C1orf158	6	3.415105	0.007369
ZNF264	6	3.193597	0.003192
ALOX5	6	0.200669	0.024066
IFFO1	6	1.981091	0.047718
MIA	6	3.272932	0.045768
